# Quantifying and mapping the burden of human and animal rabies in Iraq

**DOI:** 10.1371/journal.pntd.0008622

**Published:** 2020-10-22

**Authors:** Mashair Z. Ismail, Najlaa K. AL- Hamdi, Ali N. AL- Amery, Denise A. Marston, Lorraine McElhinney, Emma Taylor, Victor del Rio Vilas, Thani M. Dadan, Anthony R. Fooks, Daniel L. Horton

**Affiliations:** 1 Central Veterinary Laboratory, Veterinary Directorate, Baghdad, Iraq; 2 Animal and Plant Health Agency (APHA), Rabies and Viral Zoonoses Group, (WHO Collaborating Centre for the Characterisation of Rabies and Rabies-Related Viruses, OIE Reference Laboratory for Rabies), Weybridge, New Haw, Surrey, United Kingdom; 3 University of Liverpool, Institute of Infection & Global Health, Liverpool, United Kingdom; 4 University of Surrey, School of Veterinary Medicine, Guildford, United Kingdom; 5 Center for Disease Control, Zoonosis Section, Ministry of Health, Baghdad, Iraq; 6 University of London, St George's Hospital Medical School, Institute for Infection and Immunity, London, United Kingdom; US Department of Agriculture, UNITED STATES

## Abstract

Rabies was first reported in ancient Iraqi civilizations, yet it remains a poorly quantified and important public health threat in the region. Efforts to control rabies in Iraq including dog population control, and vaccination of livestock and dogs, have increased since 2010. Officially reported data on human rabies, dog bites, and animal rabies cases between 2012 and 2017 are analysed here to assess the effect of existing control efforts, to inform future strategies, and to highlight gaps in surveillance and reporting. The results of molecular characterization of 32 viruses from animal cases from throughout Iraq are presented, to improve the understanding of rabies dynamics in the animal reservoir. Although annual numbers of reported human cases were lower in the period between 2012 and 2017 than prior to 2010, human cases continue. There was a distinct gender and age bias among human cases with nine cases in males for every one female and twice as many cases in children than adults. Spatial clustering analysis and phylogenetic evidence suggests rabies is endemic throughout the country, with no regional variation in risk, but better surveillance and reporting is required to underpin control strategies.

## Author summary

Rabies was first reported from the region that is modern day Iraq, yet the disease remains a poorly quantified and important public health threat there. Efforts to control rabies in Iraq including culling and vaccinating dogs, have been increased since 2010, but their effect on the disease is unknown. Officially reported data on human rabies, reported dog bites, and animal rabies cases between 2012 and 2017 are analysed here to assess the effect of existing control efforts, to inform future strategies, and to highlight gaps in surveillance and reporting. The results of virus typing of 32 viruses from animal cases from throughout Iraq are presented, to improve understanding of which strains of the virus are circulating in the country. Results of the analysis show that although annual numbers of reported human cases were lower after 2012 than prior to 2010, human cases continue. There was a distinct gender and age bias among human cases with nine cases in males for every one in females and twice as many cases in children than adults. Investigating the distribution of cases among governates, and reconstructing the evolutionary history of the viruses using virus typing data suggests rabies is endemic throughout the country, with no differences in risk between areas. These data together provide useful information for prioritizing control efforts, but demonstrate that better surveillance and reporting is required.

## Introduction

Rabies is a fatal zoonotic viral disease that can infect all mammalian species including humans. First reported in ancient Iraqi civilizations 4000 years ago, it remains one of the most feared zoonotic threats in the Middle East region including Iraq [1–3]. The majority of rabies cases are caused by rabies virus, one of 16 recognised species of RNA viruses in in the genus *Lyssavirus*, within the family *Rhabdoviridae* [4, 5].

Of the estimated annual 59,000 human deaths worldwide more than 30,000 are thought to occur in Asia [6, 7]. At least 99% of these human cases are attributed to bites of infected dogs which act as the main reservoir for the virus in most regions [8–10]. However wildlife has a notable impact on the maintenance and the transmission of rabies virus and in many areas both dog and wildlife rabies occur at the same time [2, 11, 12]. In Iraq, free roaming dogs are considered the main reservoir for maintaining the virus, however, rabies is also reported in wildlife, mainly in the western area of the country and in neighboring countries [2]. Since 2003, ongoing conflicts have had an impact on disease incidence, and control in Iraq [13]. Official health reports demonstrated an increase in human rabies cases countrywide, particularly in Baghdad, with a threefold increase between 2001 and 2010 [1]. Molecular characterization of animal rabies virus strains from Baghdad indicated the viruses belonged to a single variant and were closely related to rabies viruses from Turkey, Syria, Iran and Georgia [1].

Since 2010, efforts to control rabies in Iraq have increased. Initially campaigns supported by the regional police were organized to eliminate stray dogs. More recently, efforts for rabies control were undertaken by the veterinary departments through vaccination of livestock and owned dogs. The officially reported data on human rabies cases and reported dog bite incidence were reviewed to assess the effect of commissioned intervention strategies. The results of animal rabies sampling, and molecular characterization of viruses from a wider geographic area of Iraq than previous studies are also described, to improve understanding of rabies dynamics in the animal reservoir.

## Methods

### Ethics statement

The study was assessed by the University of Surrey Self-Assessment Governance and Ethics (SAGE) process for human (514292-514283-64999342) and animal (631851-631842-65051923) research. It was not possible to seek consent for the analysis of the human rabies data as the data were de-identified.

### Human rabies data

Human suspect rabies cases between 2012 and 2017 and reported dog bite records between 2012 and 2016 were reviewed (S1 STROBE checklist). Reported human rabies cases (de-identified, but including district of origin, patient gender and age), and numbers of patients presenting with a history of dog bite (district of origin only) were obtained with permission from the Zoonosis Section of the Center for Disease Control (CDC), Iraqi Ministry of Health, in Baghdad. The public health offices in each of 18 provinces record and send these data to the CDC. As there is no confirmatory laboratory diagnosis for human rabies in Iraq, human case data consist of patients with a history of animal bite and clinical presentation in line the with WHO’s case definition of acute encephalitis (dominated by forms of hyperactivity or paralytic syndromes progressing towards coma and death within 7–10 days after the first symptom, if no intensive care is instituted) [7]. Reporting of suspect human rabies cases is required by law.

### Animal rabies sampling

Fifty eight frozen brains of rabies suspect animals (livestock and dogs), with a history of clinical signs consistent with rabies, were submitted to the Central Veterinary Laboratory (CVL) in Baghdad from veterinary offices throughout Iraq during the period 2013–2017. These submissions were the result of either one of the private veterinary clinics, government veterinarians, or animal owners reporting suspicion of rabies to one of the 230 the local government veterinary offices. A suspected case was defined according the OIE definition of a susceptible animal that shows a change in behavior followed by death within ten days, or that displays any of the following signs: hypersalivation, paralysis, lethargy, abnormal aggression or abnormal vocalization. An additional two hundred dog brain samples were selected randomly from frozen stored samples from a campaign for stray dog culling in 15 provinces in Iraq during 2013 and 2014.

All laboratory work to characterize the rabies virus strains was undertaken in Iraq. All brain samples were tested using standard fluorescent antibody test (FAT) for lyssavirus antigen [14]. The 58 rabies suspect animals were tested at the time of submission, and the two hundred culled dog brain samples were frozen at the time of submission and tested in batches. Total RNA was extracted using RNeasy Lipid Tissue Mini Kit (Qiagen) according to the manufacturer’s protocol. The first RT- PCR round described by [15] was performed using One Step RT-PCR kit (Qiagen). A second round hemi-nested RT-PCR was applied using Top Tag Master Mix kit (Qiagen) according to the manufacturer’s instructions. PCR products were purified using Gel/PCR DNA Fragments Extraction kit (Geneaid) following the manufacturer’s instructions. All amplicons were sequenced using chain-termination (Sanger) sequencing by an ABI-3730XL capillary machine (Macrogen Inc., Seoul, Gyeonggi-do, South Korea). Two primers were used (forward and reverse primers of the second round PCR). Sequence comparison and analyses were performed using DNAstar package (Lasergene) and MEGA6 software package [16]. The optimum substitution model was determined using Modeltest to be the Kimura 2 parameter model with rate variation among sites (K2+G). A maximum likelihood tree was generated using MEGA6 comparing the sequences from Iraq to those from surrounding countries.

### Data analysis

De-identified human rabies data were analysed for differences in age and gender with Chi-squared tests, using expected gender and age group frequencies from previous studies (1). Both human and animal rabies data were assessed for geographic and temporal trends, and tested for spatial autocorrelation between provinces using Moran’s index.

## Results

### Human rabies surveillance

The total number of reported suspect rabies cases was 54 (S1 Fig), with an annual average of 9 (SD 4.7). The highest human rabies count was in 2016 when there were 17 cases, equating to 0.05 cases per 100,000 population (using a total population estimate of 37 million [17]). Gender and age data were available for years 2013–2017 (n = 52), showing a distinct gender bias with nine cases in males for every one female case (p<0.001) and a total of 38 cases in children under 15 years old (73%) and only 14 cases in >15 year olds (27%) (p<0.001) (S1 Table, S2 Table).

During the period between 2012 and 2016, there was an average of 12,358 (SD 2368) dog bites reported in Iraq annually (S2 Fig). This is equivalent to approximately 33 reported bites per 100,000 population every year.

### Geospatial analysis

**Fig 1:**
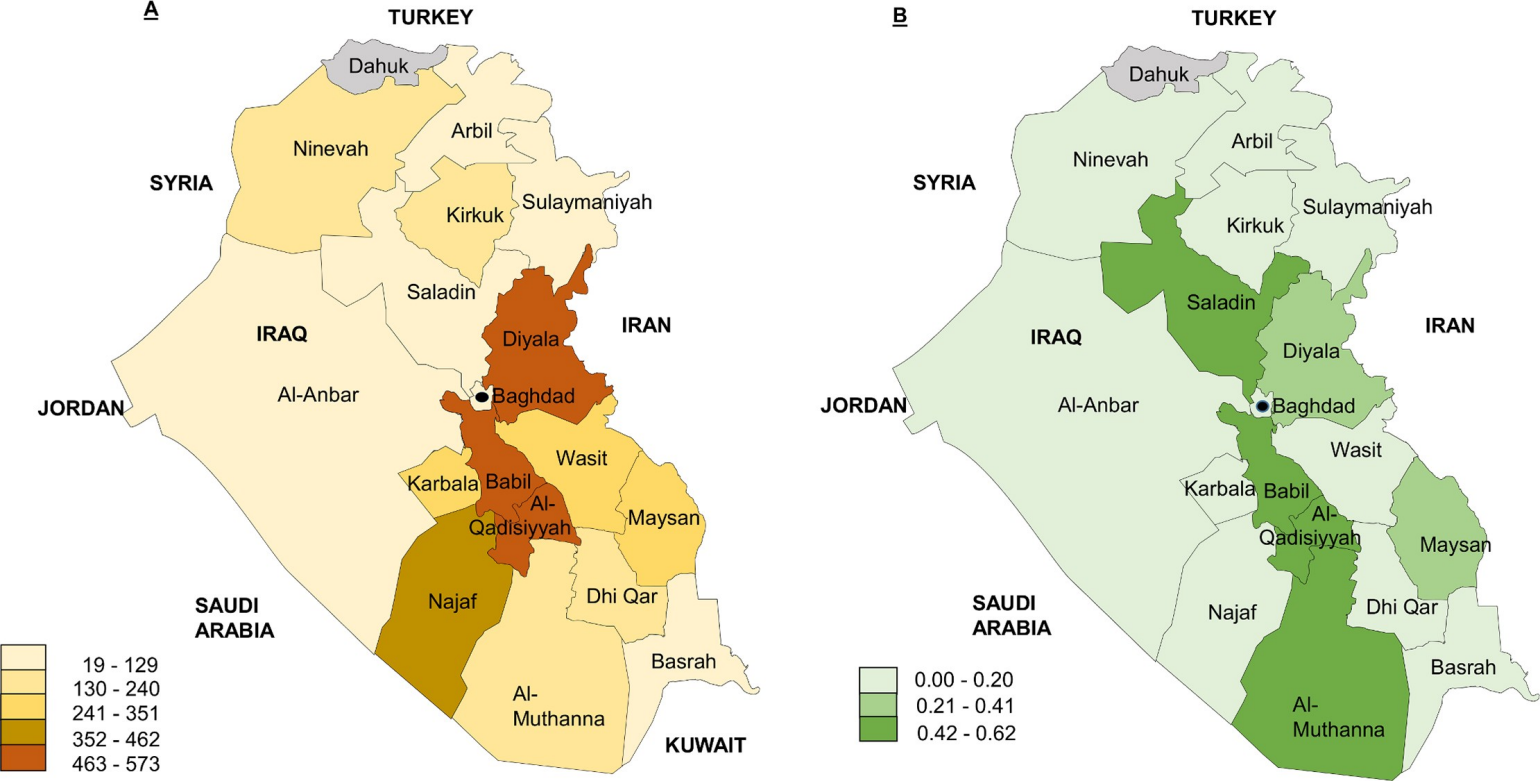
**A**. Reported dog bites per 100,000 population between 2012 and 2016 across 18 provinces in Iraq, and **B.** Reported human rabies cases per 100,000 population between 2012 and 2017 across 18 provinces in Iraq. Provinces are colored in groups clustered using natural breaks in the data. (grey = no data).

Human suspect rabies cases appear widely distributed throughout the country (Fig 1A and 1B). Twelve out of 18 provinces reported human suspect rabies cases, distributed from the northern to southern extremes of the country. Cumulative incidence in those provinces varies from 0.05 to 0.70 cases per 100,000 population [18], but there is no evidence for geographic clustering (Moran’s I statistic = -0.16, p = 0.95). Spatial analysis of reported dog bite rates demonstrated four provinces in the south of the country with higher bite rates than all others, but no evidence of significant geographic clustering (Moran’s I statistic -0.04, p = 0.86). The remaining 14 provinces had consistently lower reported bite rates but several provinces (Al-Muthanna, Maysan and Salahdin) had high counts of human cases despite lower reported bite rates.

### Animal rabies sampling

**Fig 2:**
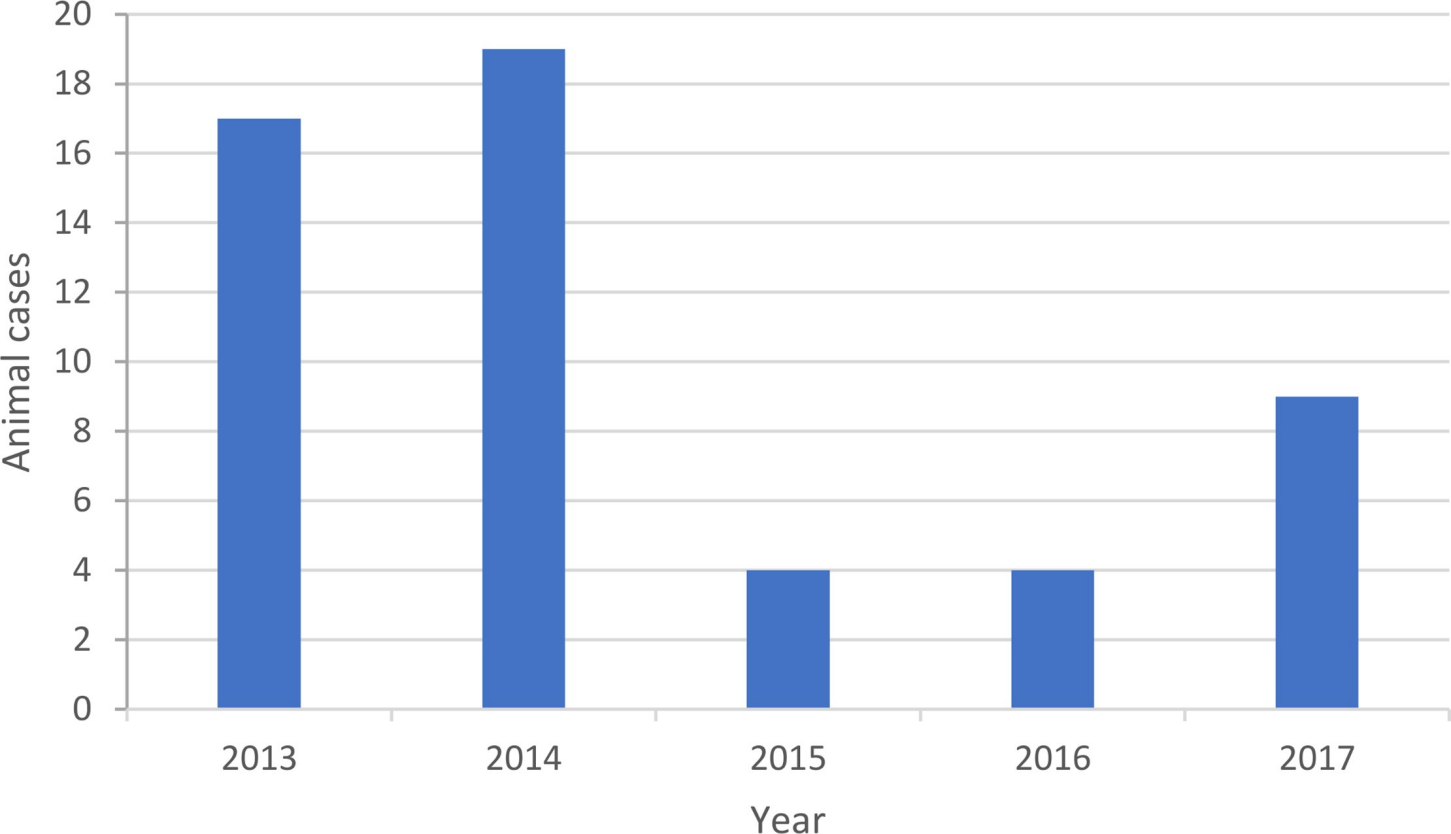
Total laboratory confirmed animal rabies cases in Iraq from 2013–2017.

Fifty three out of 58 (91%) of samples from rabies-suspect animals (38 cattle and 15 dogs) were rabies virus positive when tested by FAT during the period (2013–2017) (Fig 2). In the three years between 2015 and 2017 the annual average of reported animal cases was five (SD = 3.54) cases, compared to an average of 18 (SD = 0.118) cases during the period 2013–2014.

In addition to rabies suspect animals, a further two hundred dogs were selected for testing from a free-roaming dog culling programme in 2013–14. Nineteen (9.5%) of these samples were positive for rabies.

**Fig 3:**
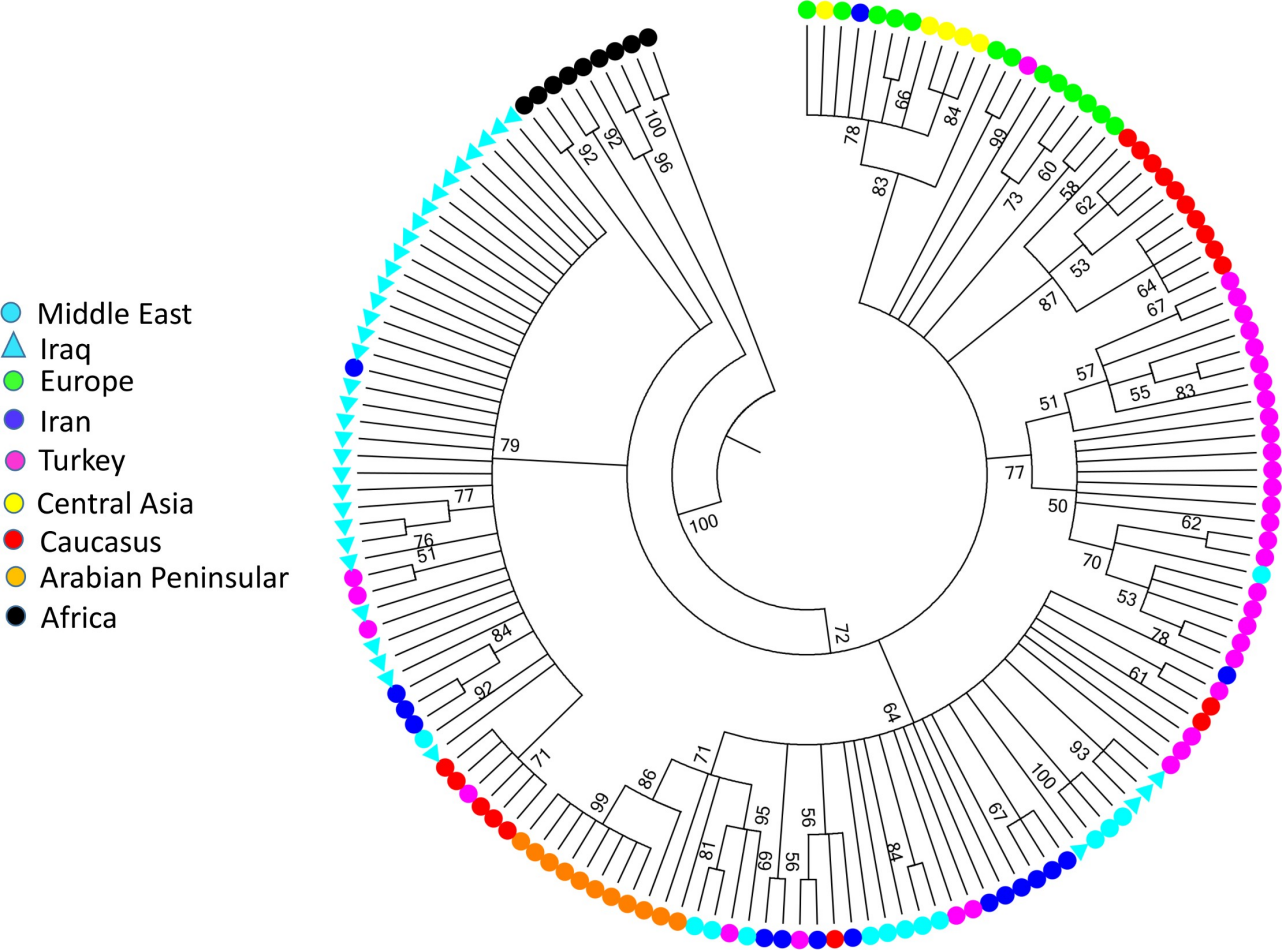
Consensus maximum likelihood phylogenetic tree using the Kimura 2-parameter model with rate variation among sites (K2+G) implemented in MEGA6. Bootstrap percentages (1000 replicates) are shown.

Thirteen out of 53 positive reported cases (8 cattle, 5 dog) in addition to the nineteen from the campaign for stray dog culling were sequenced for phylogenetic analyses. Sequence analysis confirmed that all 32 samples were rabies positive when compared to equivalent GenBank sequences. Phylogenetic relationships of new sequences from Iraq generated in this study (Fig 3) demonstrated that they were closely related to, and shared a common ancestor with, samples previously sequenced from Iraq. Sequence details are included in S3 Fig. Of the two clades previously detected in Iraq only one is represented in these new sequences (from 2013 onward) despite these samples originating from a wide area of Iraq.

## Discussion

Rabies has been a great public health concern in the Middle East region, including Iraq, for decades. In the present study, we investigated the burden of human and animal rabies. There were fewer reported human suspect rabies cases in the period between 2012 and 2017, than there were prior to the onset of concerted control efforts in 2010. The annual average number of human suspected rabies cases per year 2012–2017 was lower, at 9, than the average 2005–2010 (22, SD 4.05). Even the peak incidence in the current study, (0.05 deaths per 100,000 in 2016) was lower than in 2009 (0.09 deaths per 100,000) [19]. In Baghdad, there has been a marked decrease in the number of reported suspect cases with an average of one case per year between 2015 and 2016 compared to 6 cases per year between 2009 and 2010 [2]. These changes appear unrelated to population size, which is estimated to have grown consistently at a rate of approximately 2% during the period of study [17]. These apparent reductions in suspect cases after 2010 are encouraging and may be related to the increase control efforts at the time but the downward trend does not appear to be continuing, with relatively steady numbers of suspect cases during this study period (Fig 1). The predominance of males and children among suspect cases concurs with previous studies in Iraq, and other regions [1] and strongly supports targeted public awareness campaigns. Despite differences in reported numbers of suspect cases between provinces, these cases were reported throughout the country and there was no evidence of geographical clustering. Although reporting of suspect cases is obligatory by law, we cannot rule out reporting bias between provinces.

The administration of rabies immunoglobulin and vaccines could limit the possibility of human deaths due to rabies if given in a timely manner and in accordance with WHO guidelines. Post exposure prophylaxis (PEP) is provided free of charge in Iraq, in line with the WHO category of exposure. However, insufficient public knowledge and low risk awareness may reduce the number of individuals completing the vaccination course. Accurate records of PEP uptake and course completion are not available but would help inform strategies to improve compliance and save lives [20].

The control of rabies in dog populations is an important target with an internationally agreed goal to eliminate dog-mediated human rabies by 2030 [21–23]. Surveillance is essential to assess the elimination and control efforts in rabies control programmes and to quantify the burden of disease to prioritize resources [24]. Our present study included sampling from animals culled as part of a national control programme, to provide an estimation of animal rabies prevalence in free roaming dogs. Our results showed a proportion of these dogs were positive for rabies (9.5% of 200 sampled). Although clinical data are not available for these culled dogs, this proportion is similar to that in found-dead dogs in rabies endemic regions [25] but higher than that previously reported for apparently healthy dogs in other endemic regions. For example, a maximum of 5% positive was demonstrated in Nigeria, and researchers could not confirm the health status of the animals [26]. The reason for the apparently high proportion of rabid dogs among the culled sample is not known but culling process may have been biased toward aggressive or sick dogs. Of the suspect animal rabies cases submitted to the CVL for the period 2013–2017, the highest number of positive cases (41% of the total) was from Babel. This high number of animal cases coincided with higher numbers of human suspect rabies cases.

There were temporal trends in the numbers of animal cases. In the three years 2015–2017 the annual average of reported animal cases was lower than the period 2013–2014 but this is based on a small sample size and should be interpreted with caution. Reporting was ad-hoc and dependent on awareness and vigilance of owners and veterinarians. During the period of the study the mean number of veterinarians and veterinary paraprofessionals reported to the OIE was 5150 (range 2337–11130) [27]. Furthermore, most of the animal cases reported were cattle, (38/53,71%). Infected cattle are considered a ‘dead-end’ host of rabies, not contributing to onward transmission. The phylogenetic relatedness showed rabies sequences obtained from infected cattle and dogs were in the same lineage in all provinces tested. This concurs with a previous study in Iraq and demonstrates that dogs are the most likely source of bovine rabies [1]. However, wildlife were under-represented in the samples submitted to CVL for testing and therefore the role of wildlife in the maintenance and transmission of rabies in Iraq remains unclear until further wildlife surveillance is undertaken. Rabies cases in livestock are frequently associated with wildlife in other countries in the region [28].

Molecular epidemiology enables an understanding of the evolutionary relationships of rabies viruses detected in animals [2, 29]. Our present study provided an overview of the circulating rabies virus in dogs and livestock (cattle) in Iraqi provinces. All of the sequences from viruses collected as part of this study, share a common ancestor with viruses from Syria, Turkey and Iran suggesting their common origin. Close phylogenetic relatedness when compared with those from surrounding countries and the high similarity among sequences detected over 18 provinces and a ten year period in Iraq, suggest that rabies virus is being maintained in Iraq. Two strains previously reported as unique to Iraq (Acc. no. KP723603&KP723604.1) [1] were not detected in the current study although analyses such as these are highly susceptible to bias in the host species tested in part due to the reliance on a proactive veterinary infrastructure to collect, transport and submit samples for testing. The partial genome studies presented here have limited resolution but demonstrate the feasibility of virus characterization using in-country expertise and infrastructure. Further studies with more viruses represented, including those from wildlife, and longer sequences are required to reliably infer the geographic and temporal spread of rabies in the region.

Despite the intense and on- going conflicts since 2003, rabies control efforts have been made in Iraq, especially in the last three years. Comprehensive campaigns which involved inter-sectoral collaboration, human, environmental, and veterinary health authorities supported by the regional police troops were organized to eliminate stray dogs [1]. However, culling is not a long term solution to eliminate rabies in Iraq [30]. More efforts for rabies elimination were conducted by the veterinary departments during the last two years through vaccination of livestock and owned dogs, although coverage levels are unknown. Although levels of human suspect rabies are lower than previously reported, the data presented here suggest that the target for elimination of dog mediated human rabies by 2030 will require further enhancement of rabies control in the region. A centralized animal and human rabies diagnosis laboratory, combined with obligatory notification of rabies cases and education on responsible pet ownership and vaccination are all recommended to enhance progress toward the control of rabies in Iraq.

## Supporting information

S1 Checklist STROBE checklist. (DOCX)

S1 Fig The number of human rabies cases in Iraq, reported to the Zoonosis Section of the Center for Disease Control (CDC), Iraqi Ministry of Health, between 2012 and 2017. (PDF)

S2 Fig The number of dog bites reported to the Zoonosis Section of the Center for Disease Control (CDC), Iraqi Ministry of Health, between 2012 and 2016. (PDF)

S3 Fig Consensus maximum likelihood phylogenetic tree using the Kimura 2-parameter model with rate variation among sites (K2+G) implemented in MEGA6. Bootstrap percentages (1000 replicates) and sequence identities are shown.
(PDF)

S1 Table Reported human rabies cases by gender. (DOCX)

S2 Table Reported human rabies cases by age. (DOCX)
