# Supplementary material for: Quantifying and mapping the burden of human and animal rabies in Iraq
Source: PLoS Negl Trop Dis. 2020 Oct 22;14(10):e0008622. doi: 10.1371/journal.pntd.0008622 (PMC7580899; doi:10.1371/journal.pntd.0008622)
Supplement: S1 Checklist — (DOCX) [file pntd.0008622.s001.docx]

STROBE Statement—checklist of items that should be included in reports of observational studies

|  | Item No. | Recommendation | Page  No. | Relevant text from manuscript |
| --- | --- | --- | --- | --- |
| **Title and abstract** | 1 | (*a*) Indicate the study’s design with a commonly used term in the title or the abstract | 1 | Quantifying and mapping the burden of human and animal rabies in Iraq |
|  |  | (*b*) Provide in the abstract an informative and balanced summary of what was done and what was found | 2 | Rabies was first reported in ancient Iraqi civilizations, yet it remains a poorly quantified and important public health threat in the region. Efforts to control rabies in Iraq including dog population control, and vaccination of livestock and dogs, have been implemented since 2010. Officially reported data on human rabies, reported dog bites, and animal rabies cases between 2012 and 2017 are analysed here to assess the effect of existing control efforts, to inform future strategies, and to highlight gaps in surveillance and reporting. The results of molecular characterization of 32 viruses from animal cases from throughout Iraq are presented, to improve the understanding of rabies dynamics in the animal reservoir. Although annual numbers of reported human cases were lower in the period between 2012 and 2017 than prior to 2010, human cases continue. There was a distinct gender and age bias among human cases with nine cases in males for every one female and twice as many cases in children than adults. Spatial clustering analysis and phylogenetic evidence suggests rabies is endemic throughout the country, with no regional variation in risk, but better surveillance and reporting is required to underpin control strategies. |
| Introduction | | | |  |
| Background/rationale | 2 | Explain the scientific background and rationale for the investigation being reported | 4 | Rabies is a fatal zoonotic viral disease that can infect all mammalian species including humans. First reported in ancient Iraqi civilizations 4000 years ago, it remains one of the most feared zoonotic threats in the Middle East region including Iraq |
| Objectives | 3 | State specific objectives, including any prespecified hypotheses | 5 | Since 2010, efforts to control rabies in Iraq have increased. Initially campaigns supported by the regional police were organized to eliminate stray dogs. More recently, efforts for rabies control were undertaken by the veterinary departments through vaccination of livestock and owned dogs. The officially reported data on human rabies cases and reported dog bite incidence were reviewed to assess the effect of commissioned intervention strategies. The results of animal rabies sampling, and molecular characterization of viruses from a wider geographic area of Iraq than previous studies are also described, to improve understanding of rabies dynamics in the animal reservoir. |
| Methods | | | |  |
| Study design | 4 | Present key elements of study design early in the paper | 5 | The officially reported data on human rabies cases and reported dog bite incidence were reviewed to assess the effect of commissioned intervention strategies. The results of animal rabies sampling, and molecular characterization of viruses from a wider geographic area of Iraq than previous studies are also described, to improve understanding of rabies dynamics in the animal reservoir. |
| Setting | 5 | Describe the setting, locations, and relevant dates, including periods of recruitment, exposure, follow-up, and data collection | 6 | Human rabies data:  Human rabies cases between 2012 and 2017 and reported dog bite records between 2012 and 2016 were reviewed. Reported human rabies cases (including district of origin, patient gender and age), and numbers of patients presenting with a history of dog bite (district of origin only) were obtained from the Zoonosis Section of the Center for Disease Control (CDC), Iraqi Ministry of Health, in Baghdad. The regional public health offices in each of 18 provinces record these data then send to the CDC. As there is no confirmatory laboratory diagnosis for human rabies cases in Iraq the diagnosis is based on the history of dog bite, human clinical symptoms and death of the patient. |
| Participants | 6 | (*a*) *Cohort study*—Give the eligibility criteria, and the sources and methods of selection of participants. Describe methods of follow-up  *Case-control study*—Give the eligibility criteria, and the sources and methods of case ascertainment and control selection. Give the rationale for the choice of cases and controls  *Cross-sectional study*—Give the eligibility criteria, and the sources and methods of selection of participants | 6 | As there is no confirmatory laboratory diagnosis for human rabies cases in Iraq the diagnosis is based on the history of dog bite, human clinical symptoms and death of the patient. |
|  |  | (*b*) *Cohort study*—For matched studies, give matching criteria and number of exposed and unexposed  *Case-control study*—For matched studies, give matching criteria and the number of controls per case |  | n/a |
| Variables | 7 | Clearly define all outcomes, exposures, predictors, potential confounders, and effect modifiers. Give diagnostic criteria, if applicable | 8 | Human rabies data were analysed anonymously for differences in age and gender. Both human and animal rabies data were assessed for geographic and temporal trends, and tested for spatial autocorrelation using Moran’s index. |
| Data sources/ measurement | 8* | For each variable of interest, give sources of data and details of methods of assessment (measurement). Describe comparability of assessment methods if there is more than one group |  |  |
| Bias | 9 | Describe any efforts to address potential sources of bias | 8 | data were assessed for geographic and temporal trends, and tested for spatial autocorrelation using Moran’s index. |
| Study size | 10 | Explain how the study size was arrived at | 6 | Human rabies cases between 2012 and 2017 and reported dog bite records between 2012 and 2016 were reviewed. Reported human rabies cases (including district of origin, patient gender and age), and numbers of patients presenting with a history of dog bite (district of origin only) were obtained from the Zoonosis Section of the Center for Disease Control (CDC), Iraqi Ministry of Health, in Baghdad. |

Continued on next page

| Quantitative variables | 11 | Explain how quantitative variables were handled in the analyses. If applicable, describe which groupings were chosen and why | 9 | Provinces are colored in groups clustered using natural breaks in the data. |
| --- | --- | --- | --- | --- |
| Statistical methods | 12 | (*a*) Describe all statistical methods, including those used to control for confounding | 8 | Human rabies data were analysed anonymously for differences in age and gender with Chi-squared tests, using expected gender and age group frequencies from previous studies (1). Both human and animal rabies data were assessed for geographic and temporal trends, and tested for spatial autocorrelation using Moran’s index. |
|  |  | (*b*) Describe any methods used to examine subgroups and interactions |  | n/a |
|  |  | (*c*) Explain how missing data were addressed |  | n/a |
|  |  | (*d*) *Cohort study*—If applicable, explain how loss to follow-up was addressed  *Case-control study*—If applicable, explain how matching of cases and controls was addressed  *Cross-sectional study*—If applicable, describe analytical methods taking account of sampling strategy | n/a |  |
|  |  | (*e*) Describe any sensitivity analyses |  | n/a |
| Results | | | | |
| Participants | 13* | (a) Report numbers of individuals at each stage of study—eg numbers potentially eligible, examined for eligibility, confirmed eligible, included in the study, completing follow-up, and analysed | 8 | The total number of reported rabies cases was 54… Gender and age data were available for years 2013-2017 (n=52), |
|  |  | (b) Give reasons for non-participation at each stage | n/a |  |
|  |  | (c) Consider use of a flow diagram |  |  |
| Descriptive data | 14* | (a) Give characteristics of study participants (eg demographic, clinical, social) and information on exposures and potential confounders | 8 | Gender and age data were available for years 2013-2017 (n=52), during which there was a distinct gender bias among cases with nine cases in males for every one female case (p<0.001) and a total of 38 cases in children under 15 years old (73%) and only 14 cases in >15 year olds (27%) (p<0.001) (Tables S4 and S5). |
|  |  | (b) Indicate number of participants with missing data for each variable of interest |  |  |
|  |  | (c) *Cohort study*—Summarise follow-up time (eg, average and total amount) |  |  |
| Outcome data | 15* | *Cohort study*—Report numbers of outcome events or summary measures over time | *8* | The total number of reported rabies cases was 54 (Figure S1), with an annual average of 9 (SD 4.7). The highest human rabies count was in 2016 when there were 17 cases, equating to 0.05 cases per 100,000 population (using a total population estimate of 37 million (17)). Gender and age data were available for years 2013-2017 (n=52), during which there was a distinct gender bias among cases with nine cases in males for every one female case (p<0.001) and a total of 38 cases in children under 15 years old (73%) and only 14 cases in >15 year olds (27%) (p<0.001) (Tables S4 and S5). |
|  |  | *Case-control study—*Report numbers in each exposure category, or summary measures of exposure |  |  |
|  |  | *Cross-sectional study—*Report numbers of outcome events or summary measures |  |  |
| Main results | 16 | (*a*) Give unadjusted estimates and, if applicable, confounder-adjusted estimates and their precision (eg, 95% confidence interval). Make clear which confounders were adjusted for and why they were included | 9 | Twelve out of 18 provinces reported human rabies cases, distributed from the Northern to Southern extremes of the country. Cumulative incidence in those provinces varies from 0.05 to 0.70 cases per 100,000 population (18), but there is no evidence for geographic clustering (Moran’s I statistic =-0.16, p=0.95). Spatial analysis of reported dog bite rates demonstrated four provinces in the south of the country with higher bite rates than all others, but no evidence of significant geographic clustering (Moran’s I statistic -0.04, p=0.86). The remaining 14 provinces had consistently lower reported bite rates but several provinces (Al-Muthanna, Maysan and Salahdin) had high counts of human cases despite lower reported bite rates. |
|  |  | (*b*) Report category boundaries when continuous variables were categorized |  |  |
|  |  | (*c*) If relevant, consider translating estimates of relative risk into absolute risk for a meaningful time period |  |  |

Continued on next page

| Other analyses | 17 | Report other analyses done—eg analyses of subgroups and interactions, and sensitivity analyses |  |  |
| --- | --- | --- | --- | --- |
| Discussion | | | | |
| Key results | 18 | Summarise key results with reference to study objectives | 12 | In the present study, 18 provinces of Iraq were investigated to assess the burden of human and animal rabies. There were fewer reported human rabies cases in the period between 2012 and 2017, than there were prior to the onset of concerted control efforts in 2010. The annual average number of human rabies cases per year 2012-2017 was lower, at 9, than the average 2005-2010 (22, SD 4.05). |
| Limitations | 19 | Discuss limitations of the study, taking into account sources of potential bias or imprecision. Discuss both direction and magnitude of any potential bias | 13  15 | Accurate records of PEP uptake and course completion are not available but would help inform strategies to improve compliance and save lives  …..although analyses such as these are highly susceptible to surveillance bias in part due to the reliance on a proactive veterinary infrastructure to collect, transport and submit samples for testing. The partial genome studies presented here have limited resolution but demonstrate the feasibility of virus characterization using in-country expertise and infrastructure. Further studies with more viruses represented, including those from wildlife, and longer sequences are required to reliably infer the geographic and temporal spread of rabies in the region. |
| Interpretation | 20 | Give a cautious overall interpretation of results considering objectives, limitations, multiplicity of analyses, results from similar studies, and other relevant evidence | 16 | Although levels of human rabies are lower than previously reported, the data presented here suggest that the target for elimination of dog mediated human rabies by 2030 will require further enhancement of rabies control in the region. |
| Generalisability | 21 | Discuss the generalisability (external validity) of the study results | 15 | Further studies with more viruses represented, including those from wildlife, and longer sequences are required to reliably infer the geographic and temporal spread of rabies in the region. |
| Other information | |  | | |
| Funding | 22 | Give the source of funding and the role of the funders for the present study and, if applicable, for the original study on which the present article is based | 16 | DLH was part funded by an Academy of Medical Sciences Springboard Award supported by the Wellcome Trust |

*Give information separately for cases and controls in case-control studies and, if applicable, for exposed and unexposed groups in cohort and cross-sectional studies.

**Note:** An Explanation and Elaboration article discusses each checklist item and gives methodological background and published examples of transparent reporting. The STROBE checklist is best used in conjunction with this article (freely available on the Web sites of PLoS Medicine at http://www.plosmedicine.org/, Annals of Internal Medicine at http://www.annals.org/, and Epidemiology at http://www.epidem.com/). Information on the STROBE Initiative is available at www.strobe-statement.org.
