# Supplementary figures and images for: Quantifying and mapping the burden of human and animal rabies in Iraq
Source: PLoS Negl Trop Dis. 2020 Oct 22;14(10):e0008622. doi: 10.1371/journal.pntd.0008622 (PMC7580899; doi:10.1371/journal.pntd.0008622)

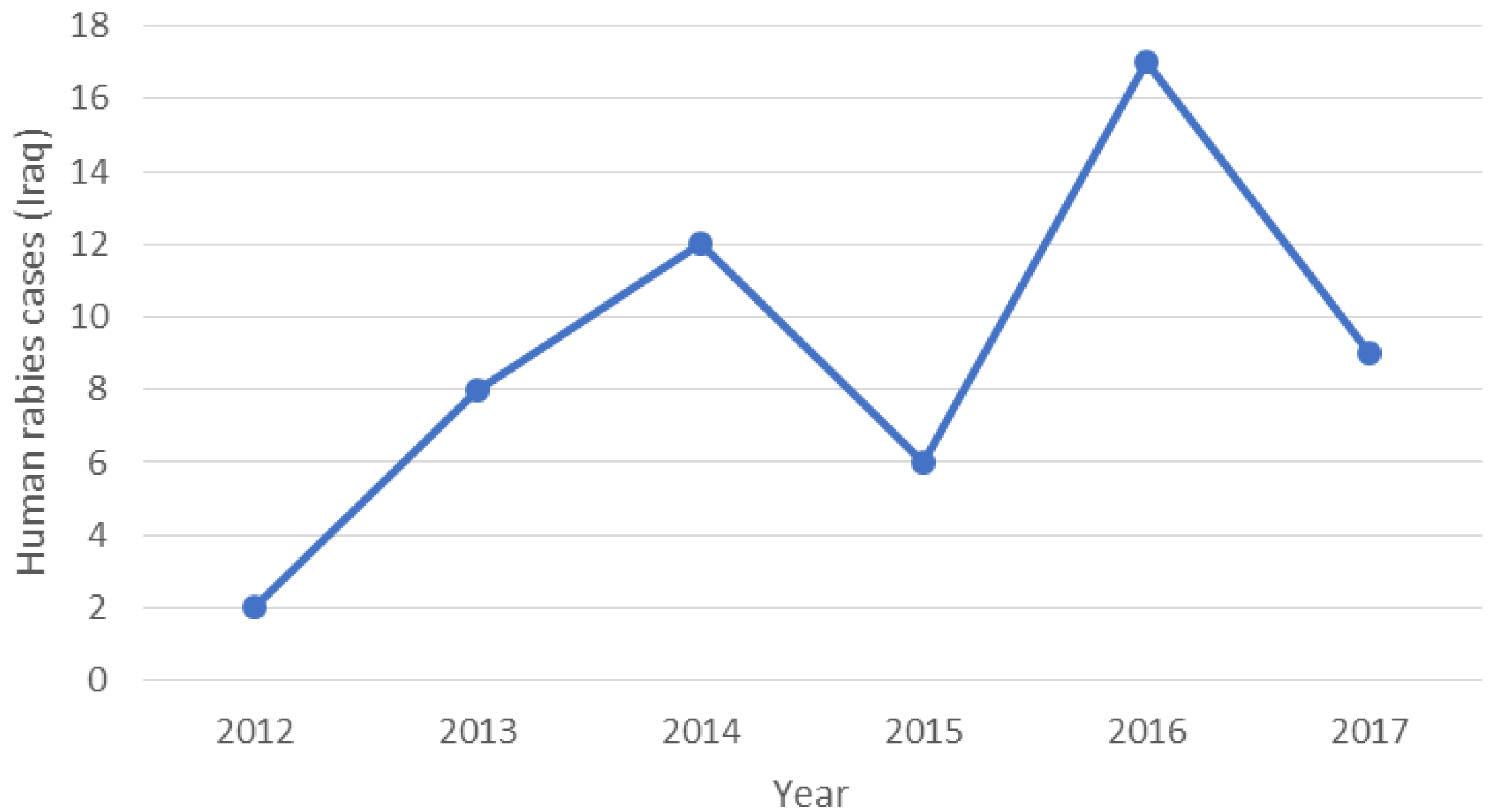

Supplement: S1 Fig — (PDF) [file pntd.0008622.s002.pdf]

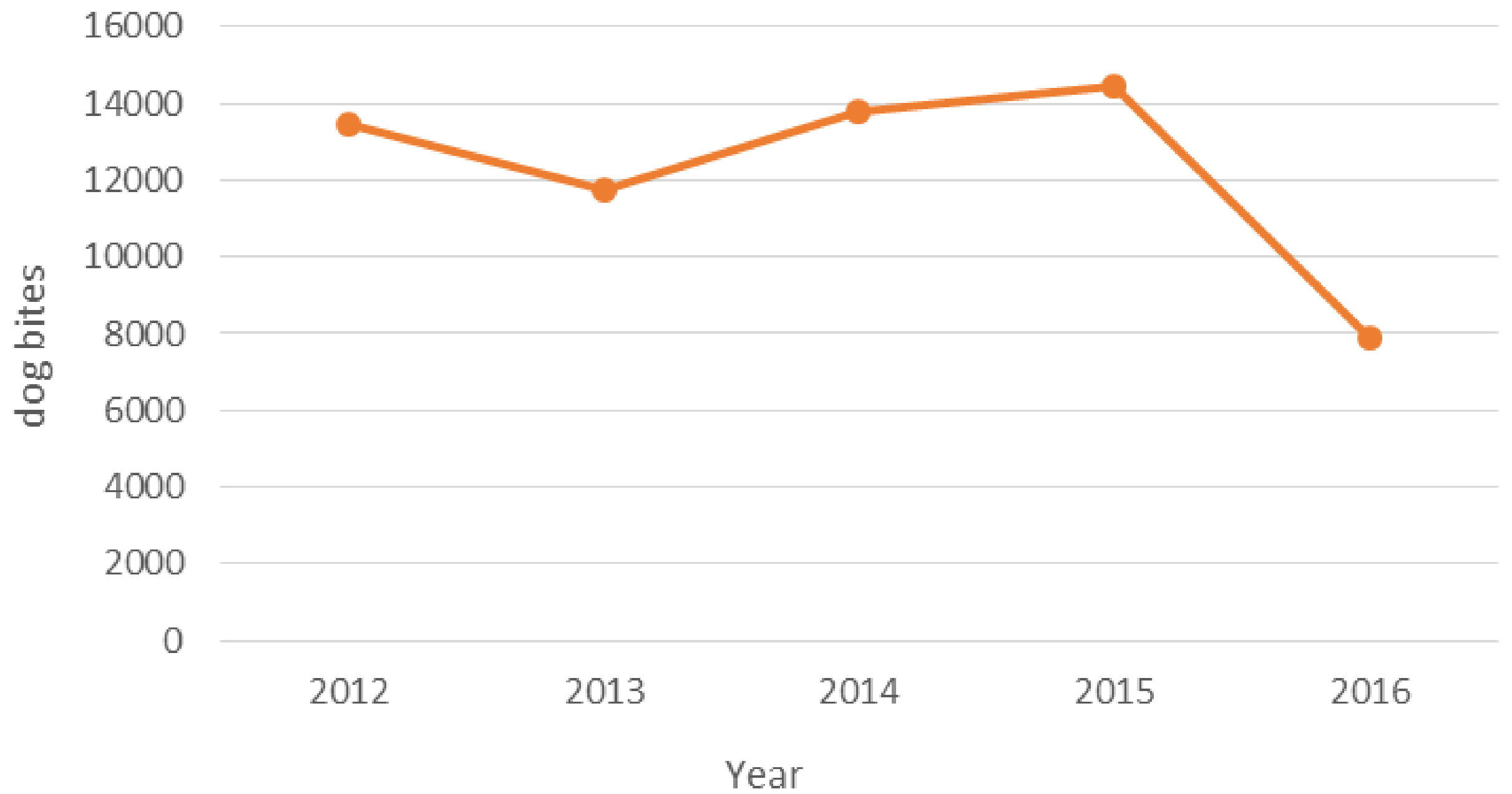

Supplement: S2 Fig — (PDF) [file pntd.0008622.s003.pdf]

- Middle East
- ▲ Iraq
- Europe
- Iran
- Turkey
- Central Asia
- Caucasus
- Arabian Peninsular
- Africa

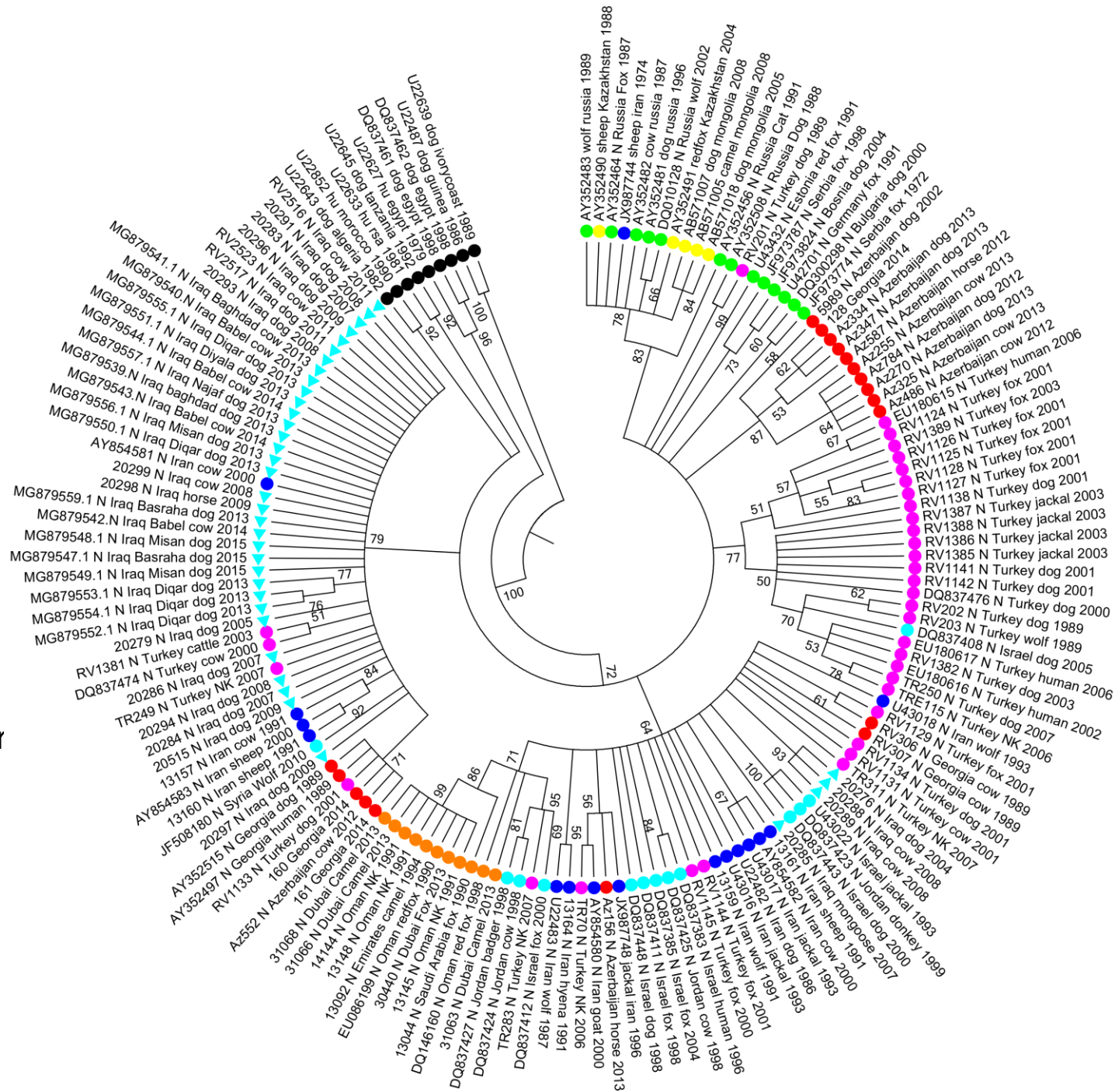

Supplement: S3 Fig — Bootstrap percentages (1000 replicates) and sequence identities are shown. (PDF) [file pntd.0008622.s004.pdf]
