# Supplementary material for: Quantifying and mapping the burden of human and animal rabies in Iraq
Source: PLoS Negl Trop Dis. 2020 Oct 22;14(10):e0008622. doi: 10.1371/journal.pntd.0008622 (PMC7580899; doi:10.1371/journal.pntd.0008622)
Supplement: S1 Table — (DOCX) [file pntd.0008622.s005.docx]

**S4 Table Human rabies cases according to age (2013-2017)**

| Year | Age (0- 15] | Age ( 15- ) |
| --- | --- | --- |
| 2013 | 5 | 3 |
| 2014 | 9 | 3 |
| 2015 | 4 | 2 |
| 2016 | 13 | 4 |
| 2017 | 7 | 2 |
