# Supplementary material for: Quantifying and mapping the burden of human and animal rabies in Iraq
Source: PLoS Negl Trop Dis. 2020 Oct 22;14(10):e0008622. doi: 10.1371/journal.pntd.0008622 (PMC7580899; doi:10.1371/journal.pntd.0008622)
Supplement: S2 Table — (DOCX) [file pntd.0008622.s006.docx]

**S5 Table. Human rabies cases according to gender (2013-2017)**

| Year | Male | Female |
| --- | --- | --- |
| 2013 | 7 | 1 |
| 2014 | 10 | 2 |
| 2014 | 5 | 1 |
| 2016 | 16 | 1 |
| 2017 | 9 | - |
